# Tip cross-sectional geometry predicts the penetration depth of stone-tipped projectiles

**DOI:** 10.1038/s41598-020-70264-y

**Published:** 2020-08-06

**Authors:** Jase Sitton, Brett Story, Briggs Buchanan, Metin I. Eren

**Affiliations:** 1 Department of Civil and Environmental Engineering, Lyle School of Engineering, Southern Methodist University, Dallas, TX 75205 USA; 2 Department of Anthropology, University of Tulsa, Tulsa, OK 74104 USA; 3 Department of Anthropology, Kent State University, Kent, OH 44242 USA; 4 Department of Archaeology, Cleveland Museum of Natural History, Cleveland, OH 44106 USA

**Keywords:** Anthropology, Archaeology, Cultural evolution

## Abstract

Understanding prehistoric projectile weaponry performance is fundamental to unraveling past humans’ survival and the evolution of technology. One important debate involves how deeply stone-tipped projectiles penetrate a target. Theoretically, all things being equal, projectiles with smaller tip cross-sectional geometries should penetrate deeper into a target than projectiles with larger tip cross-sectional geometries. Yet, previous experiments have both supported and questioned this theoretical premise. Here, under controlled conditions, we experimentally examine fourteen types of stone-tipped projectile each possessing a different cross-sectional geometry. Our results show that both tip cross-sectional area (TCSA) and tip cross-sectional perimeter (TCSP) exhibit a strong, significant inverse relationship with target penetration depth, although TCSP’s relationship is stronger. We discuss why our experimental results support what is mathematically predicted while previous experiments have not. Our results are consistent with the hypothesis that when stone tip cross-sectional geometries become smaller over time in particular contexts, this evolution may be due to the selection of these attributes for increased penetration.

## Introduction

Killing prey was vital for the survival of prehistoric humans, and projectile penetration depth contributes to the killing of prey^1–9^. A wound that is shallow is more likely to result in hunting failure relative to one that injures a critical internal organ (^10^: 84). If a projectile has to penetrate roughly into the middle of the preys’ chest cavity to achieve a lethal wound^11^, then penetration depths of 20–25 cm would be required for large ungulates (^7^: 203;^12,13^: 60;^14^: 554, but see^16^: 86), and depths of over 50 cm would be required for even larger animals like bison (^14^: 554). If an animal’s critical organs are not injured, then a more deeply penetrating projectile will possess a better chance of causing severe blood loss or a blood trail that can be tracked^16^. However, it is important to note that there are cases where projectile tips serve as delivery devices for poison, which may negate the need for deep penetration^17^. It is also important to note that the penetration lethality estimates reported above need further scientific validation and study. Nevertheless, regardless of the absolute penetration depth needed for a lethal wound, there is agreement that increasing projectile penetration would be a boon to prehistoric people.

Given the importance of increasing projectile penetration depth for ensuring successful hunts when poison is not employed, it is reasonable to conclude that prehistoric people in particular contexts selected for projectile weapon system characteristics that would have improved this factor. While there are many potential variables that can influence stone-tipped projectile penetration depth, two often mentioned are tip cross-sectional area (TCSA) and tip cross-sectional perimeter (TCSP) (e.g.^3,5–7,9,18–26^). Following Hughes^5^, these variables are defined as

$${{\rm TCSA}}=\frac{1}{2}{w}_{tip}{t}_{tip}$$

and

$${{\rm TCSP}}=4\sqrt{{\left(\frac{{w}_{tip}}{2}\right)}^{2}+{\left(\frac{{t}_{tip}}{2}\right)}^{2}}$$

where ${w}_{tip}$ and ${t}_{tip}$ are the width and thickness, respectively, of the point measured at the widest location on the point. Given that Sperrazza and Kokinakis^27^, Ashby^28^, Hughes^5^, and Kneubuehl^29^ mathematically demonstrate target penetration is inversely proportional to tip cross-sectional area, then, all things being equal, stone-tipped projectile penetration depth should increase as TCSA and TCSP decrease (see also^6,19^).

Despite this theoretical foundation, experiments involving prehistoric replica stone tipped projectiles have not consistently supported the inverse relationship between penetration depth and TCSA or TCSP. For example, Salem and Churchill’s^7^ comparison of wood- versus stone-tipped projectiles (the former possessing half the TCSA of the latter) was consistent with the mathematical predictions, as was Mika et al.’s^6^ recent assessment of small triangular stone arrow tips. However, Wood and Fitzhugh’s^9^ experiments assessing the penetration depths of bone, stone biface, and stone microblade points—all possessing different TCSA and TCSP values—showed no significant difference in penetration depth. Sisk and Shea’s (^24^: 2044) analysis of triangular (Levallois) points showed no relationship between TCSA and penetration depth (r = −0.080, *p* > 0.05) and only a weak inverse relationship between TCSP and penetration depth (r = −0.242, *p* < 0.01). Perhaps most critical of the relationship between TCSA and TCSP with penetration depth is Clarkson^3^. His ballistics experiments involving stone projectile tips possessing a large range of TCSA and TCSP values led him to suggest that “a very poor correlation exists between these two statistics and penetration depth” (^3^: 193) such that “TCSA/TCSP also do not provide a valuable measure of the mechanical limits on projectile design” (^3^: 197).

Given the disparate conclusions between theoretical predictions and experimental results, and the contrasting verdicts between different sets of experiments, we conducted a ballistics experiment that explicitly assessed whether TCSA and TCSP were robust indicators of projectile penetration potential.

## Materials and methods

### Stone points

**Figure 1:**
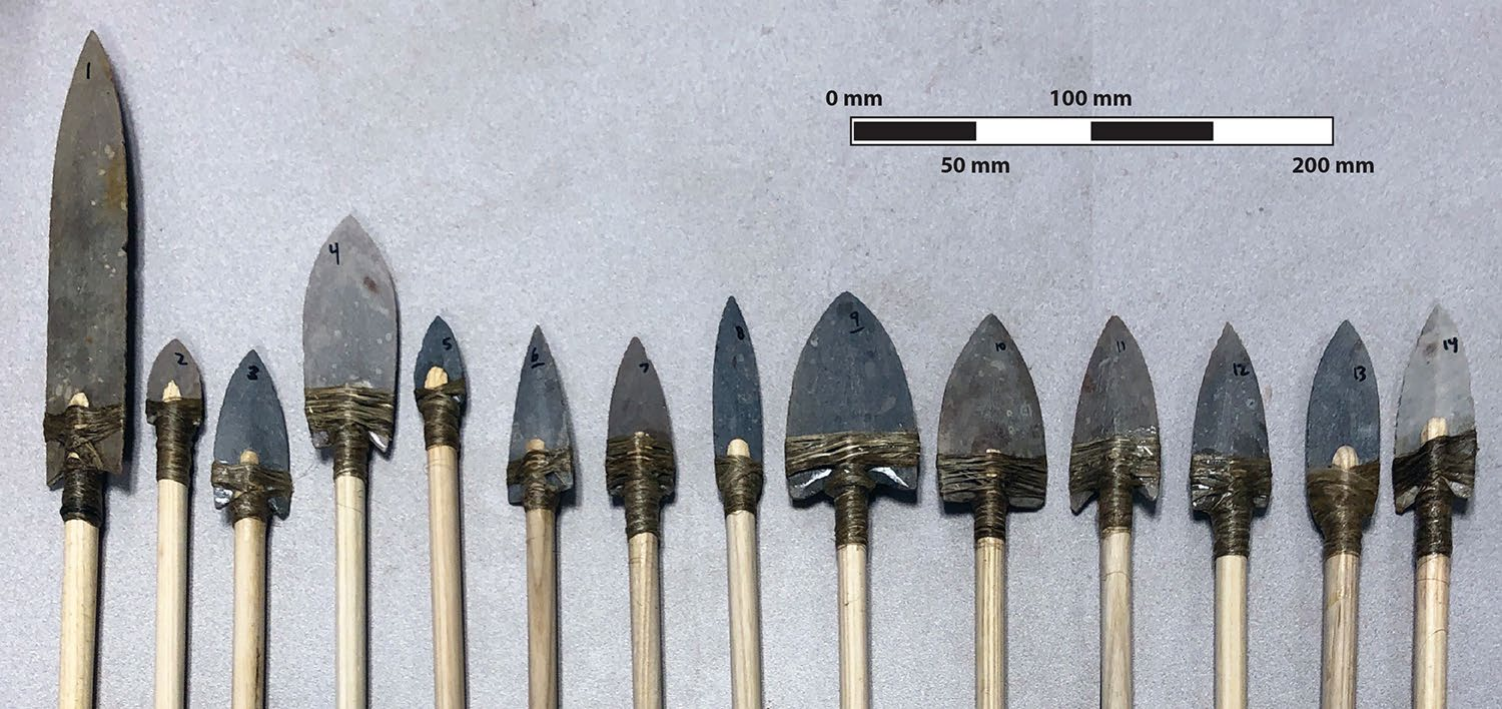
The fourteen hafted point forms; the specimen designations begin on the left with point 1 and finish on the right with point 14 (see Table 1).

Fourteen forms of lanceolate stone projectile point were produced by Neolithics Flintknapping Supply House (www.neolithics.com) using Texas Fredericksburg chert (Fig. 1; Table 1). First, slabs of chert were cut out with a rock saw, put in a kiln, and heat-treated to 450°. Then a pattern for each particular point shape was drawn on the slabs and cut out with a trim saw. Next, each point shaped slab was rough ground with a 30-grit diamond wheel followed by a 60-grit diamond wheel to produce a typical stone tip’s lenticular shape.

### Hafting

The projectiles were hafted to ½ in. (1.27 cm) ash dowels manufactured by Thunderbird Atlatl (www.thunderbirdatlatl.com) (Fig. 1). The dowels were milled to fit the various sizes of stone points. Hemp fiber and Kodak gelatin based glue dissolved in warm water was used for hafting the projectiles on to the dowels. A small electric heated glue pot was used to maintain the correct viscosity of the glue. The method for attaching the stone points was to first shape the wood to fit the point. The wood and the stone points were dipped into the glue pot. Next, a measured amount of fiber was dipped into the glue pot. The glue was spread evenly on the fiber and then wrapped over the wood/stone joint by hand. Care was taken to make sure that there was a good connection free from voids. The glue was allowed to dry for 24 h, inspected, and then packaged for delivery.

### Experimental procedures

Our experimental procedures followed closely the procedures described in other ballistics studies conducted at The Kent State University Experimental Archaeology Laboratory (^6^; 30–34). The hafted specimens were shot with a compound bow (29 lbs. draw weight) mounted in the Spot-Hogg “Hooter-Shooter” in a controlled indoor setting.

The distance between the stationary target and bow was approximately 180 cm, which allowed adequate room for the specimens to travel once fired without drastically losing speed or dropping (^31^: 39). We fired the hafted projectile specimens into blocks of moist clay containing crystalline silica, which has been used as a substitute for meat and tissue in other studies (^32^; 35–38). The clay was terracotta low-fire earthenware clay, commonly referred to as “potter's clay” (^34^: 5,840–5,841).

Each of the fourteen projectile point forms were shot into a clay target thirty times. No damage occurred to any of the points when fired into the clay, allowing each one to be fired repeatedly. We recorded penetration depth into the clay target for each shot. We measured this variable by holding the shaft at the location at which the shaft was first exposed in the clay target (^31^: 41). Once we removed a specimen from the target, a tape measure was used to measure from the person’s finger mark on the shaft to the tip of the point. The mean penetration depth for each of the fourteen projectile point types was calculated from the 30 shots for that point type (Table 1). We made sure that no projectile entered the hole of a previous shot into clay, and we systematically kneaded and pounded the clay when there were too many holes.

**Table 1:** Data recorded on the fourteen projectiles and variables calculated for the analyses.

Point form	Projectile mass (g)	Mean velocity (m/s)	Point width (mm)	Point thickness (mm)	TCSA (mm^2^)	TCSP (mm)	KE (J)	Momentum (kg m/s)	Mean penetration depth (cm)
1	139.30	22.85	40.76	7.06	143.88	82.73	34.26	3.09	14.80
2	55.60	33.46	21.26	5.83	61.97	44.09	35.36	1.98	22.49
3	69.90	32.15	28.76	5.25	75.50	58.47	35.62	2.23	18.37
4	85.90	28.86	37.81	6.87	129.88	76.86	35.78	2.48	16.27
5	62.40	34.25	20.32	4.90	49.78	41.80	35.58	2.11	22.77
6	64.70	33.38	25.65	5.60	71.82	52.51	36.05	2.16	17.99
7	63.90	34.29	25.31	5.76	72.89	51.91	35.16	2.12	18.71
8	66.40	32.75	19.76	5.92	58.49	41.26	36.22	2.19	19.72
9	93.80	29.07	49.68	7.49	186.05	100.48	36.87	2.63	14.35
10	88.60	28.52	43.83	7.39	161.95	88.90	35.41	2.50	14.78
11	77.90	30.39	32.90	7.08	116.47	67.31	36.38	2.38	16.66
12	80.40	29.97	29.05	7.17	104.14	59.84	36.12	2.41	17.96
13	80.80	29.23	27.90	6.88	95.98	57.47	34.52	2.36	15.43
14	78.20	29.25	29.25	8.30	121.39	60.81	35.00	2.34	18.97

KE and momentum are means excluding outliers.

Each projectile was pulled to a standardized bow draw length of 48 cm. This effectively imparts a controlled, constant potential energy to the system for each test. This potential energy is converted to kinetic energy as each projectile is fired; this kinetic energy should be constant across all tests (aside from small, random experimental errors that might occur). We reasoned that a prehistoric person—given a particular weapon system—would not have been able simply to muster more energy to achieve a greater velocity with a heavier point, nor would they have necessarily used less energy to achieve a slower velocity with a lighter point. The velocities thus reflect that: given a single hypothetical individual firing all fourteen forms, the more massive projectiles travel slower than smaller ones although we note that the velocities in our experiment fall well within the range of human atlatl throwing^39^. To measure velocity (m/s), we used a Gamma Master Model Shooting Chronograph throughout the experiment (^31^: 40–41). The chronograph readings result in “error” if there is a change in sunlight, cloud cover, or some other minor variable. As a result, some of the 30 tests for each point type do not have recorded velocity values. As with penetration depth, we averaged the recorded velocities of each of the fourteen projectile point forms to produce a mean velocity (Table 1).

This mean velocity, along with projectile mass, was used to calculate the average kinetic energy and average momentum of each of the fourteen projectile point types. The kinetic energy of a single point was calculated using:

$${{\rm KE}}=\frac{1}{2}m{v}^{2}$$

where $m$ is the mass of the point and $v$ is the velocity of the point. The momentum of a single point was calculated using:

$${{\rm p}}=mv.$$

**Figure 2:**
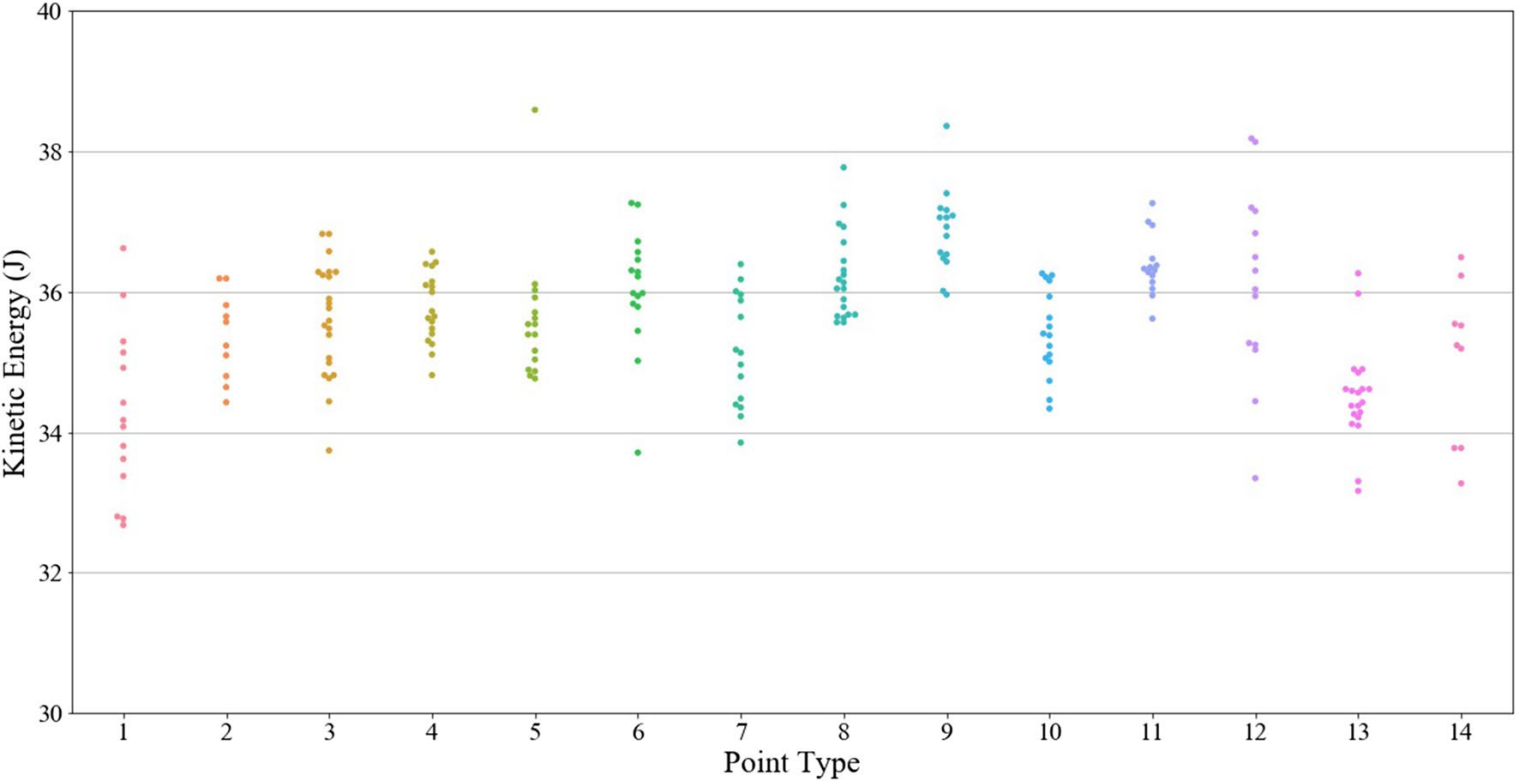
Kinetic energy by point type.

As mentioned above, kinetic energy (KE) was effectively held constant for each individual test through the use of a constant bow draw weight and draw length. KE is thus used as the control for the presented experiments and is expected to remain constant across all tests outside of small experimental errors that may occur; momentum, however, will not be constant and can thus be used as a variable in the presented statistical analyses of penetration depth. There were some instances where either the velocity of the point was unable to be measured or the kinetic energy calculated was a statistical outlier from the population of kinetic energy values. Experiments falling into either of these two categories were removed from the dataset prior to analysis. An outlier was defined as any kinetic energy value outside of 1.5IQR below the first quartile or 1.5IQR above the third quartile, where IQR is the interquartile range of the kinetic energy values. Figure 2 illustrates the consistency of the KE across most tests (statistical outliers have been removed); the average KE and standard deviation across all tests shown are 35.61 J and 1.05 J, respectively.

### Statistical assessment of penetration depth with TCSA/TCSP

TCSA and TCSP were calculated for each of the fourteen point forms (Table 1). We use a bivariate ordinary least squares (OLS) regression approach to model the relationship between the independent variables (TCSA, TCSP, and momentum) and the dependent variable (penetration depth). We checked the normality assumption for each dataset and they conformed to an underlying normal distribution (Shapiro–Wilk tests: Penetration depth W = 0.95, *p* = 0.61; TCSA W = 0.94, *p* = 0.48; TCSP W = 0.93, *p* = 0.29; Momentum W = 0.90, *p* = 0.13).

## Results

**Figure 3:**
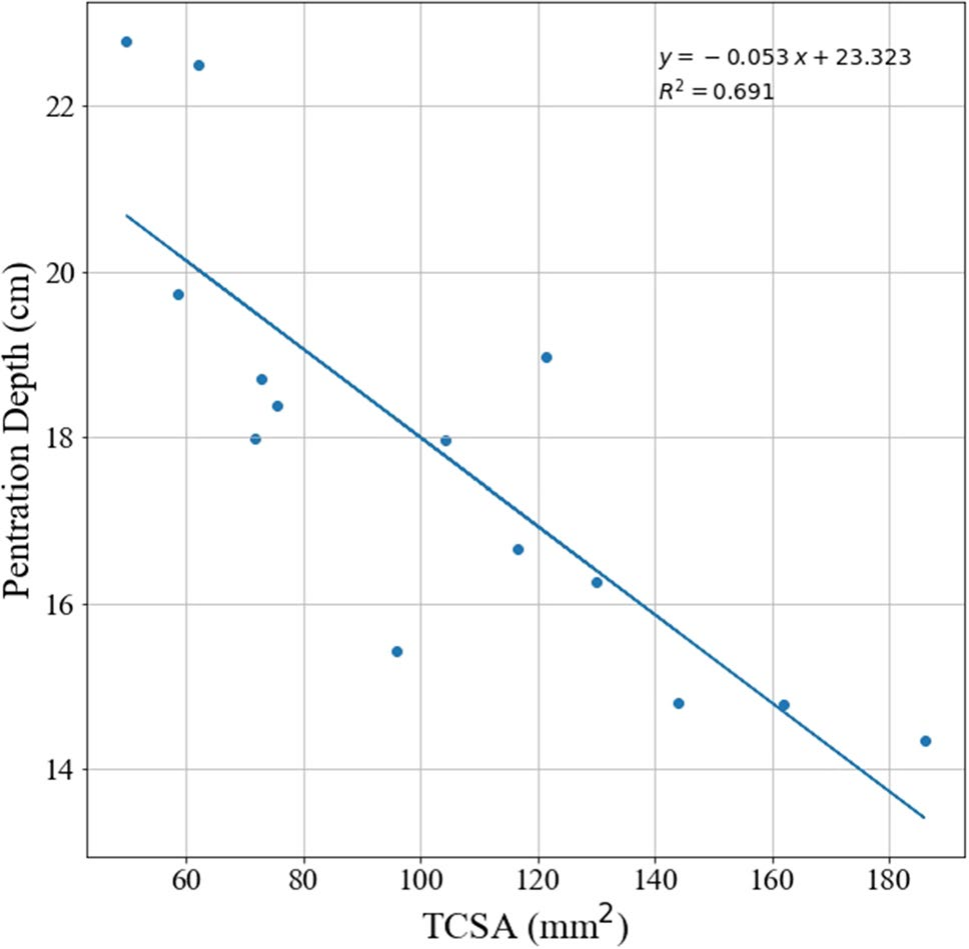
Penetration depth versus TCSA.

The OLS of TCSA predicting penetration depth of the fourteen point types has a significant negative slope (− 0.053) with an r^2^ value of 0.69, as shown in Fig. 3.

**Figure 4:**
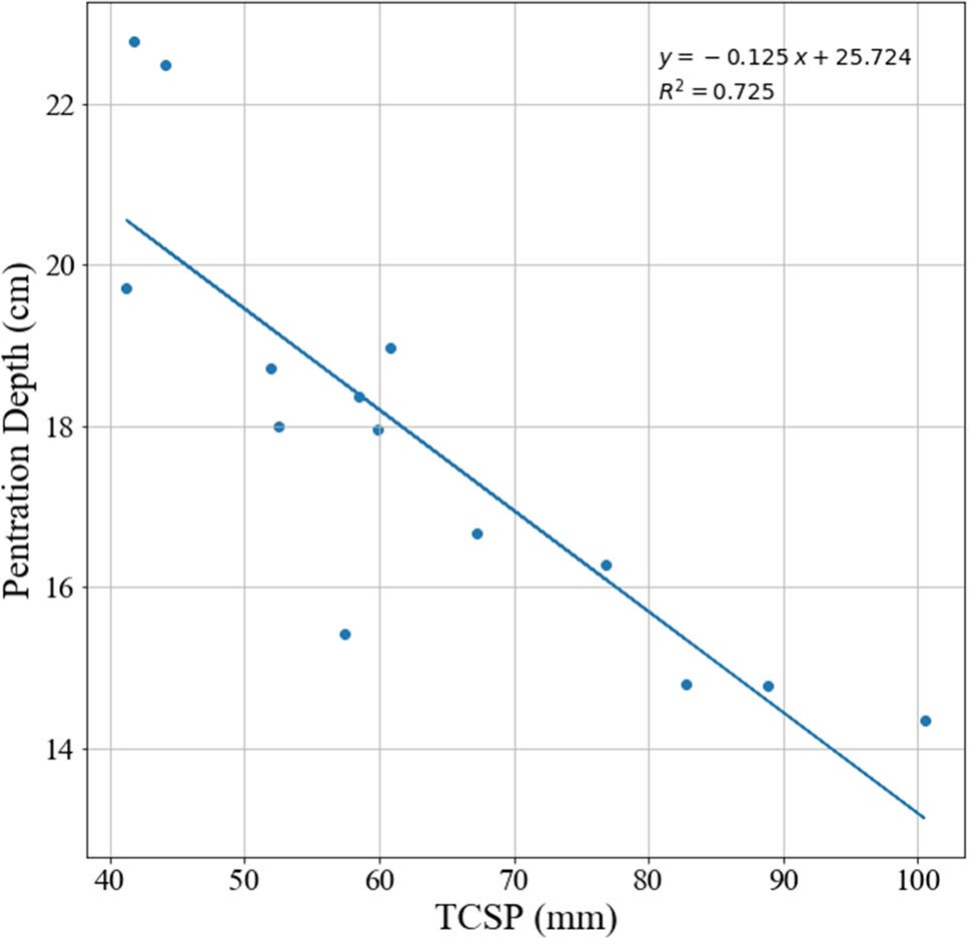
Penetration depth versus TCSP.

The OLS of TCSP predicting penetration depth also shows a significant negative relationship (slope = −0.125, r^2^ = 0.73) as shown in Fig. 4.

**Figure 5:**
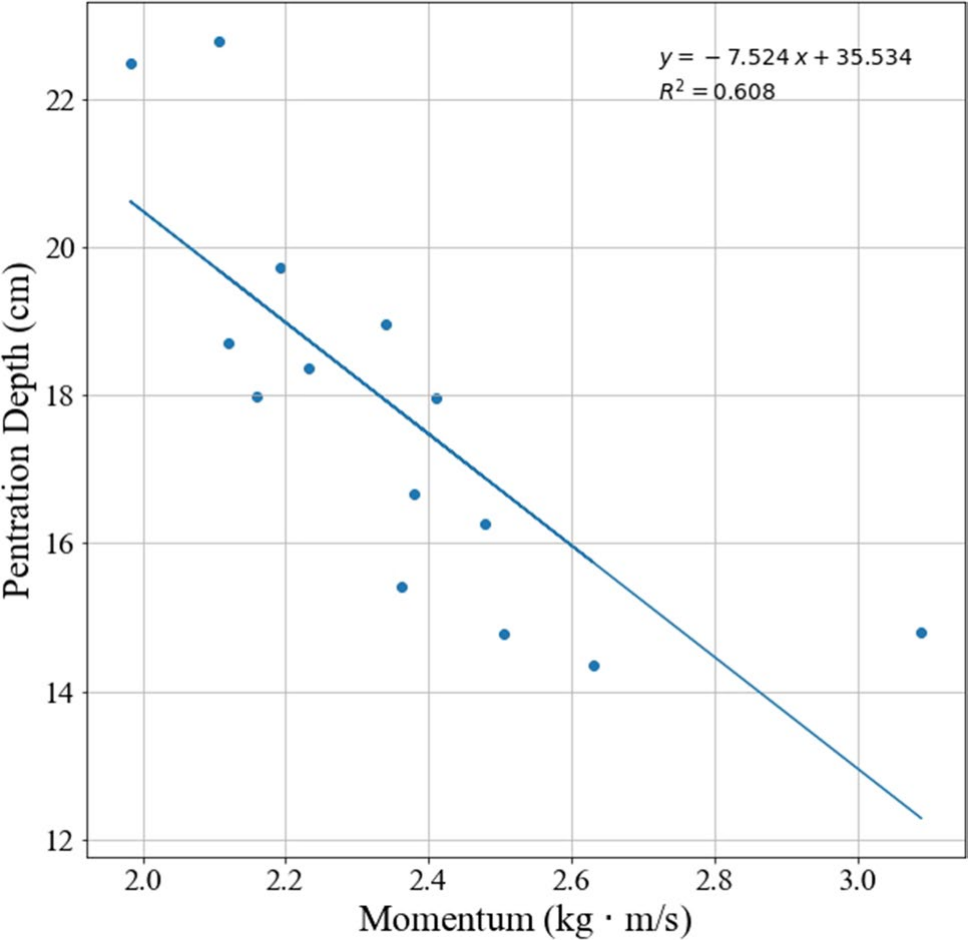
Penetration depth versus momentum.

Next, we examined the OLS relationship between momentum and penetration depth. The slope for the relationship between momentum and penetration depth is negative (− 7.524) and the variables are correlated (r^2^ = 0.61) as shown in Fig. 5.

Lastly, we entered the three models—TCSA, TCSP, and momentum—into a stepwise multiple regression model. The final model excluded TCSA and momentum as predictors and included only TCSP (adjusted r^2^ = 0.74, *df* = 1,12, F = 34.21, *p* < 0.0000).

## Discussion

Despite clear mathematical predictions for a strong inverse relationship between penetration depth and tip geometry (^5^; 27–29), recent experiments examining this relationship have yielded mixed results. In this paper, we examined the penetration depths of 14 different projectile point forms, each fired 30 times. These 30 shots per point were used to calculate a mean penetration depth for each point type. We used OLS regression analysis to investigate the relationship between this mean penetration depth and the TCSA and TCSP for each point type. Our results indicate that tip cross-sectional attributes, particularly TCSP, are robust predictors of penetration depth. In other words, these results suggest that—at velocities reasonable for stone tipped projectiles—a stone point’s cross-sectional attributes are good indicators of whether certain types of point would have potentially been more or less successful at injuring a prey’s vital organ.

We are currently unsure as to why TCSP is a more robust predictor of penetration depth than TCSA. It may have to do with the how TCSP and TCSA are calculated and how they estimate the true geometry of points. It may also have to do with the particular relationship of TCSP and TCSA in each of our 14 individual point types—perhaps assessments of other point morphologies will reveal TCSA to be a more robust indicator of penetration depth. More testing on these issues is certainly needed.

These results have important implications for the interpretation of prehistoric weaponry evolution. When archaeologists document changes in point form such that tip cross-sectional attributes become smaller over time, this evolution may be due to the selection of these attributes for increased penetration (^6^; 24). To be sure, stochastic mechanisms can also result in the decrease of tip cross-sectional attributes, thus a case for selection depends on context and must remain an inference. However, had our results shown no relationship between penetration depth and TCSA or TCSP, then the case for selection of these attributes for increasing projectile penetration depth—in any context—vanishes.

**Figure 6:**
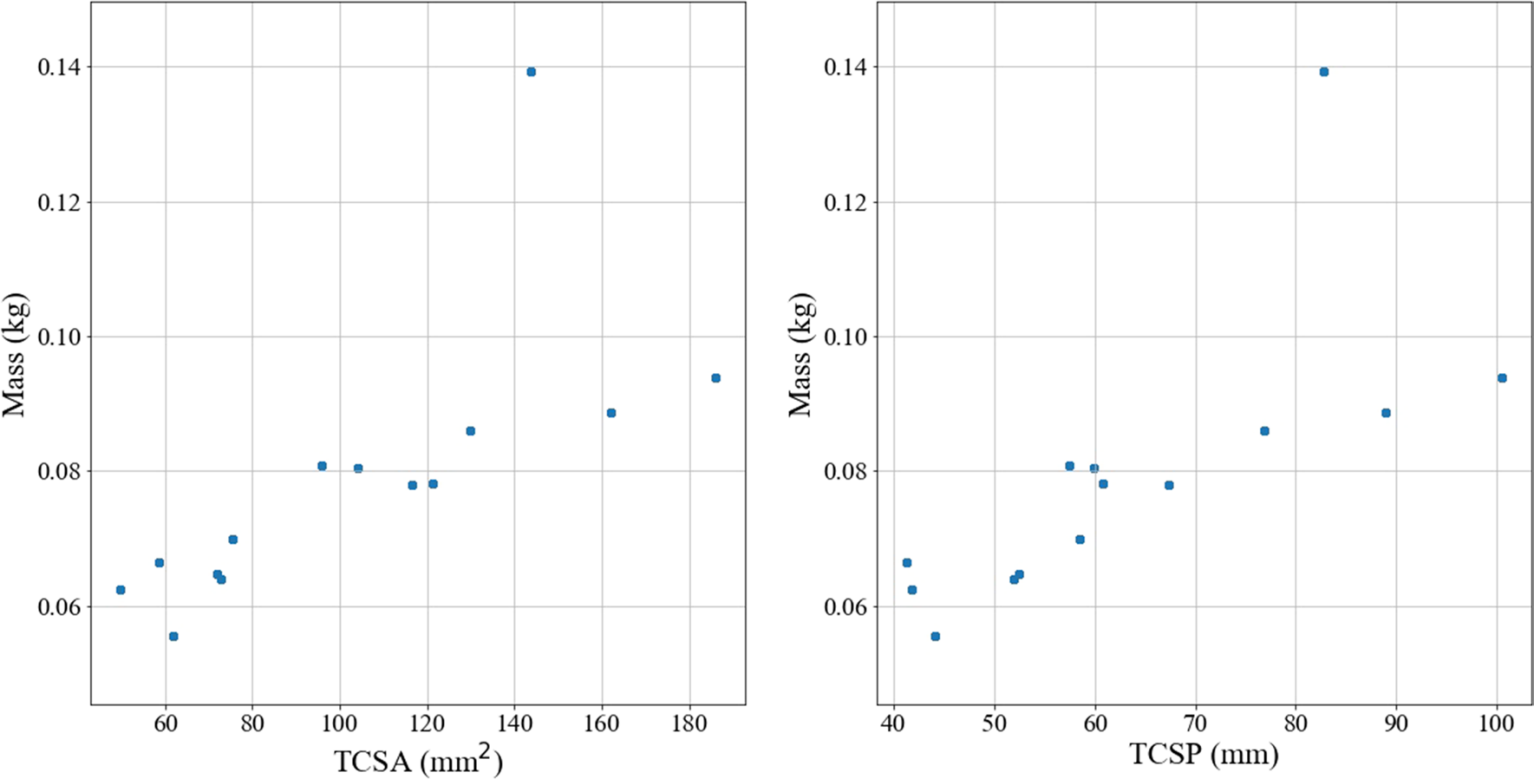
Mass versus tip geometry. “The ‘outlier’ here and in Fig. 7 is Point Type 1, which is longer than the other point types, as shown in Fig. 1. The point length is not taken into account for TCSA or TCSP. As such, Point Type 1 has a higher mass than other point types with comparable TCSA and TCSP values due to its length.”

**Figure 7:**
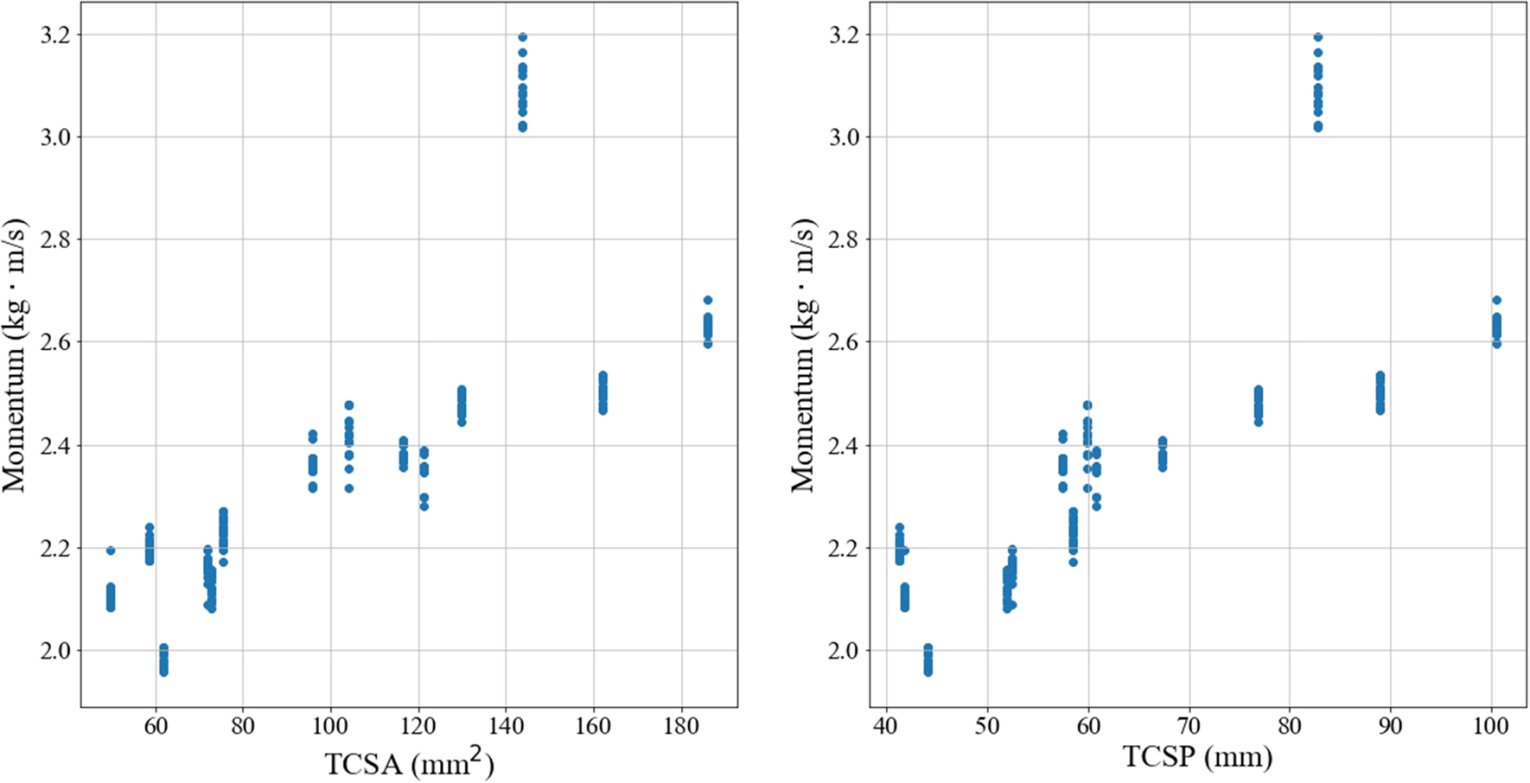
Momentum versus tip geometry. See also Fig. 6 caption.

While the OLS analysis of the relationship between penetration depth and momentum shows an inverse relationship (thus indicating that the more momentum a point has, the less likely it is to embed), the conclusion that this applies as a general rule independent of other factors cannot be drawn as momentum cannot be assumed to be an independent variable due to its dependence on the tip mass and, thus, tip geometry. As shown in Fig. 6, there appears to be a linear correlation between mass and both TCSA and TCSP. Figure 7 shows that momentum also appears to have a linear correlation with tip geometry. Point type 1 appears as an outlier in both Figs. 6 and 7; this is attributed to the fact that the point is much longer and massive than the other points and suggests that extreme geometric variations deviate from the correlations between mass (or momentum) and TCSA and TCSP.

One important question is why our results show a strong relationship between tip cross-sectional attributes while previous results of advocates and critics alike do not (^3^; 24; but see^6^). We suggest that one reason may be that the target being penetrated may have played an important role. In our experiment we used potter’s clay, a uniform substance that Key et al. (^32^: 2042) recently examined with experiments that used high-speed video analyses to verify the similarity of clay to meat when using stone points. More experiments are of course necessary to further validate the use of clay, but the use of gelatin for arrow wounds—as was done in the Clarkson^3^ study—has been questioned by Karger et al. (^40^: 499). They write “because the penetration depth in gelatin and especially in soap does not come even close to that in non­bone tissues, both media are unsuitable for experimental simulation of arrow wounds.” Furthermore, Karger et al.^40^ documented different forms of modern arrow tip behaving in opposite ways in gelatin versus meat, indicating that “field tips penetrated deeper in gelatin than in nonbone tissue but broad-heads showed the opposite behavior.” Sisk and Shea’s^24^ draping of leather over an archery target may have also added an additional variable that resulted in inconsistent penetration from shot to shot.

There could be other experimental variables accounting for the differences in the present study versus previous studies examining the relationship between tip cross-sectional attributes and penetration depth. One might be our use of the Spot Hogg Hooter-Shooter to produce consistent aim, draw length, and KE between shots. Another possible factor explaining the different results between this study and previous ones might be our use of ground points possessing only the gross morphology of bifacial points. Sisk and Shea^24^ and Clarkson^3^ used knapped points possessing flake scars that may cause more or less drag during penetration. Moreover, triangular, or minimally retouched, flakes used as point tips might possess different amounts of curvature or mass that is asymmetrically distributed across the bifacial or dorsal–ventral plane, introducing further penetration depth variation. Slight variations in the angle of penetration might be playing a large role in terms of penetration depth variation as well^7^. Finally, our averaging of thirty penetration depths per point type—where as Sisk and Shea^24^ and Clarkson^3^ only fired each of their points once—may also be contributing to the differences in this study versus those previous ones. However, if we compare our strongest predictor of penetration depth, TCSP, against all the raw penetration depth values, the correlation remains significant and strong (Spearman’s rs = −0.71, *p* < 0.0000).

In conclusion, our results strongly support the hypothesis that tip cross-sectional attributes predict penetration depth for points given the same kinetic energy. However, in spite of the fact that our results regarding TCSA/TCSP are consistent with mathematical predictions, the differences between our study and those that came before (e.g. Sisk and Shea^24^; Clarkson^3^) are substantial enough that we advocate more experimental work before the issue is considered settled.

## Supplementary information

Supplementary Information
